# Acute Respiratory Distress Syndrome Phenotypes After Stem Cell Transplantation: A Latent Class Analysis

**DOI:** 10.1097/CCE.0000000000001312

**Published:** 2025-09-05

**Authors:** Svetlana Herasevich, Kiyan Heybati, William J. Hogan, Mehrdad Hefazi, Hassan B. Alkhateeb, Zhenmei Zhang, Kelly M. Pennington, Ognjen Gajic, Carolyn Calfee, Hemang Yadav

**Affiliations:** 1 Department of Anesthesiology and Perioperative Medicine, Mayo Clinic, Rochester, MN.; 2 Department of Internal Medicine, Mayo Clinic, Rochester, MN.; 3 Division of Hematology, Mayo Clinic, Rochester, MN.; 4 Division of Pulmonary and Critical Care Medicine, Mayo Clinic, Rochester, MN.; 5 Division of Pulmonology, Critical Care, Allergy, and Sleep Medicine, Department of Medicine, University of California, San Francisco, CA.

**Keywords:** acute respiratory distress syndrome, acute respiratory failure, bone marrow transplant, hematopoietic cell transplantation, latent class analysis

## Abstract

**OBJECTIVE:**

To identify distinct phenotypes of acute respiratory distress syndrome (ARDS) developing after hematopoietic cell transplantation (HCT), using routinely available clinical data at ICU admission.

**DESIGN:**

Multicenter retrospective cohort study using latent class analysis.

**SETTING:**

ICUs across three Mayo Clinic campuses (Minnesota, Florida, and Arizona).

**PATIENTS:**

A total of 166 adult patients who developed ARDS within 120 days following HCT (96 allogeneic, 70 autologous).

**INTERVENTION:**

None.

**MEASUREMENTS AND MAIN RESULTS:**

Model selection was based on multiple metrics including Bayesian information criteria, entropy, and Vuong-Lo-Mendell-Rubin Likelihood Ratio testing. A two-class model optimally described the cohort. Class 1 (*n* = 81) was characterized by worse hypoxemia (P/F ratio 157 vs. 210, *p* = 0.002), higher Pco_2_ (41 vs. 36 mm Hg, *p* < 0.001), and higher bilirubin (1.4 vs. 0.9 mg/dL, *p* < 0.001) compared with class 2 (*n* = 85). Both classes included a mix of transplant types, transcending a simple autologous/allogeneic dichotomy, although class 1 had more allogeneic recipients (70.4% vs. 45.9%, *p* = 0.001). Although time-from-transplant was not a class-defining variable, class 1 occurred later after transplant (30.0 vs. 11.9 d, *p* < 0.001) with higher frequency of idiopathic pneumonia syndrome (14.8% vs. 2.4%, *p* = 0.004). Class 2 had more frequent neutropenia (leukocytes 0.4 vs. 5.9 × 10^9^, *p* < 0.001) and higher frequency of peri-engraftment respiratory distress syndrome (29.4% vs. 9.9%, *p* = 0.005). Outcomes were significantly worse for class 1 (90-d mortality: 72.8% vs. 48.2%, *p* = 0.001). An exploratory parsimonious model had good classification accuracy (0.90) using just six variables: leukocyte count, platelet count, bilirubin, Pco_2_, body mass index, and temperature.

**CONCLUSIONS:**

ARDS after HCT comprises two distinct phenotypes with distinct clinical characteristics and outcomes. These phenotypes align with recognized post-HCT lung injury syndromes and may reflect different underlying biological processes. This framework provides a foundation for investigating targeted therapeutic approaches.

**KEY POINTS**

**Question**: Can routinely available clinical data identify distinct phenotypes of acute respiratory distress syndrome (ARDS) developing after hematopoietic cell transplantation (HCT)?

**Findings**: In this multicenter retrospective cohort study of 166 HCT recipients with ARDS, latent class analysis identified two phenotypes with distinct clinical characteristics and significantly different 90-day mortality (73% vs. 48%). These phenotypes were not defined by transplant type alone and aligned with established post-HCT lung injury syndromes.

**Meaning**: Identifying ARDS phenotypes after HCT may enable earlier risk stratification and guide future phenotype-targeted therapies.

Hematopoietic stem cell transplantation (HCT) is a curative option for hematologic malignancies. Pulmonary complications are the largest contributors to non-relapse mortality after HCT, negatively impacting post-HCT overall survival (1–3). The most severe end of this spectrum fits under the etiology-agnostic umbrella of acute respiratory distress syndrome (ARDS), occurring in 5% of those undergoing HCT with mortality exceeding 60% (4, 5). Although overall survival following HCT has steadily improved, outcomes of those who have post-HCT respiratory failure syndromes have not (5–7). As such, there is an urgent need to better understand the post-HCT respiratory failure syndromes.

Critically ill patients represent a heterogenous population, and this is especially true of post-HCT ARDS. Post-HCT lung injury syndromes are often clinically indistinguishable at presentation. They may be infectious or noninfectious, with the latter including numerous ARDS syndromes such as peri-engraftment respiratory distress syndrome (PERDS) (8, 9), diffuse alveolar hemorrhage (DAH) (2, 10–12), or idiopathic pneumonia syndrome (IPS) (13–17). Although traditionally noninfectious, these syndromes can coexist with infection.

Over the past decade, there has been substantial interest in understanding the heterogeneity of critical care syndromes through data-driven phenotyping. The canonical example of this examined physiologic and cytokine data from ARDS clinical trials to identify two reproducible phenotypes of ARDS that have been replicated in numerous other settings (18–22). Machine learning has reproduced these cytokine-derived phenotypes using routine clinical data (23–25), which has also been used to derive latent class analysis (LCA) models de novo (26). Existing phenotyping models may not fully capture the unique pathophysiology of post-HCT ARDS, shaped by transplant-specific factors including conditioning regimens, immunosuppression, engraftment kinetics, and graft-vs.-host disease. Clustering strategies including LCA have never been studied in post-HCT ARDS.

The aim of this study was to apply clustering strategies to post-HCT ARDS, hypothesizing that routinely available clinical data from the time of ICU admission will reveal two or more distinct clusters with different clinical characteristics and trajectories.

## MATERIALS AND METHODS

### Study Design

This is a multicenter retrospective cohort study of HCT recipients at three Mayo Clinic transplant centers. The study protocol “Pulmonary complications in patients undergoing hematopoietic stem cell transplantation: a retrospective cohort study” was approved by the Mayo Clinic Institutional Review Board on May 2, 2013 (IRB number 13-002869). The requirement for written informed consent was waived by the IRB, and the study was conducted in accordance with the ethical standards of the IRB at Mayo Clinic and with the ethical principles described in the Declaration of Helsinki. This article adheres to the Strengthening the Reporting of Observational Studies in Epidemiology checklist (**Supplemental Table 1**, https://links.lww.com/CCX/B544).

### Setting and Participants

The study population consisted of consecutive adults (18 yr old or older), who underwent HCT at one of three Mayo Clinic sites: Rochester, Minnesota; Jacksonville, Florida; and Scottsdale, Arizona. Patients were included if they received HCT between January 1, 2005, and December 31, 2021, at the Minnesota campus, or between January 1, 2019, and December 31, 2021, at the Arizona or Florida campuses. These timeframes reflect data harmonization across campuses, which only occurred in 2018, making ARDS adjudication at Florida and Arizona sites infeasible before then. Patients were included if they developed ARDS within 120 days of HCT and required mechanical ventilation. ARDS ascertainment details were outlined in prior studies from this cohort (3, 5, 27, 28). Briefly, ARDS adjudication was performed by two independent investigators trained in Berlin criteria application, with disagreements resolved by a third expert reviewer. The 31 cases requiring third-party adjudication primarily involved patients where bilateral infiltrates were difficult to distinguish from cardiogenic pulmonary edema, or patients with concurrent conditions that could confound ARDS diagnosis.

### Data Retrieval

Data were retrieved from the EHR using enterprise-wide data warehouses (Mayo Data Explorer), critical care-specific warehouses (ICU DataMart), the institutional pulmonary function test database, and site-specific Hematology databases containing information on disease characteristics, chemotherapy exposures, and conditioning regimens (29, 30). Manual chart review supplemented data retrieval where needed. Prior studies have outlined the use of these databases for this and similar cohorts (3–5, 27, 28, 31–34). ARDS etiology was determined by manual chart review and classified as either infectious or noninfectious, and by presence of established post-HCT lung injury syndromes such as IPS, DAH, or PERDS (1, 2, 9, 12, 16, 35–38). Bronchiolitis obliterans syndrome was not considered due to its typical presentation later in the post-HCT course and the study’s focus on early ARDS (39–42).

### Phenotyping Strategy

LCA was used to identify clusters of post-HCT ARDS patients, consistent with prior work in ARDS phenotyping (20, 22, 43, 44). LCA is a statistical method that uncovers hidden (i.e., latent) subgroups within cohorts based on patterns in observed variables. Rather than assuming all patients belong to a single homogeneous group, LCA estimates the probability that each individual belongs to one of several unobserved subgroups, or “classes,” that explain the variability in the data. It assumes that a population’s observed distribution is composed of multiple distinct subpopulations, each with its own distribution pattern, and calculates the probability of each data point belonging to these hidden groups rather than making absolute assignments. LCA was performed using the *mclust* package (45, 46). Briefly, data were scaled and LCA was performed assuming between 1 and 6 latent classes. Final model selection was based on a combination of Bayesian Information Criteria (BIC), model stability (based on BIC sd, favoring lower sd), model complexity (favoring simplicity), parsimony index (entropy * 100/ [number of classes * log BIC]) and smallest class size (avoiding excessively small classes) (44, 47–50). BIC was used for model selection, with lower values indicating better model fit. Models were compared using the Vuong-Lo-Mendell-Rubin (VLMR) test (51–53) and classification stability was assessed using 1000 Monte Carlo simulations (54, 55). To ensure our latent class model identified the global optimum solution rather than a local optimum, we performed a sensitivity analysis with 20 different random starting points, all of which converged to identical classifications (Agreement = 100%, Kappa = 1.0, adjusted Rand index = 1.0), confirming the stability and robustness of our results.

Our approach focused on describing distinct clinical phenotypes rather than establishing causal relationships, consistent with prior exploratory phenotyping studies. Outcome analyses were intentionally unadjusted to characterize the natural history of each phenotype, recognizing that future validation studies should incorporate multivariable adjustment.

### Class-Defining Variables

Variables had to be available at the time of ICU admission and routinely collected as part of clinical care. Variables were chosen based on prior research in ARDS phenotyping (23, 26, 56) and included demographics (age, body mass index [BMI]), initial ventilator parameters (minute ventilation, tidal volume, positive end-expiratory pressure [PEEP]), arterial blood gas data (P/F ratio, Pco_2_, pH), vital signs (respiratory rate, heart rate, systolic blood pressure), and laboratory values (albumin, bicarbonate, bilirubin, creatinine, glucose, hematocrit, platelet count, leukocyte count, sodium). Transplant type (allogeneic vs. autologous) and sex were also included. A correlation matrix of candidate clustering variables was constructed (**Supplemental Fig. 1**, https://links.lww.com/CCX/B544). Time zero for class-defining variables was time of intubation. Due to high collinearity between admission bicarbonate and pH and admission tidal volume and minute ventilation, pH and minute ventilation were removed from class-defining variables. Importantly, time from HCT to ARDS development was deliberately excluded from class-defining variables to avoid creating artificially time-based clusters. Instead, it was analyzed as an outcome to determine whether naturally emerging phenotypes showed temporal patterns, which might suggest distinct biological mechanisms.

Endothelial Activation and Stress Index (EASIX) was calculated as (creatinine × lactate dehydrogenase)/platelets using pre-transplant values (57). Diffusing capacity for carbon monoxide (DLCO) was adjusted for hemoglobin.

### Statistical Analyses

JMP Pro Software (SAS Institute, Cary, NC) was used for data collection and handling. Data analysis was performed in R 4.3.3 (R Foundation for Statistical Computing, Vienna, Austria) using the R Studio 2024.03 integrated development environment (PBC, Boston, MA). Specific packages included *tidyverse*, *mice*, *mclust*, *GGally*, *survival*, and *survminer* (45, 58–60). Custom functions were created for Monte Carlo simulations, LCA metrics and the VLMR test.

Patient demographics and clinical characteristics were described using counts (percentages), means (sd), or medians (interquartile range [IQR]) as appropriate. A *t*-test, Wilcoxon Rank-Sum or chi-square test were used to determine the statistical significance for normally distributed continuous, non-normally distributed continuous and categorical variables, respectively.

Within clustering variables, missing data were handled through multiple imputation using the Multiple Imputation by Chained Equations (*mice*) package. Specific variables with missing data frequency were: albumin (23.5%), glucose (6.6%), bicarbonate level (5.4%), initial tidal volume (2.4%), initial minute ventilation (1.8%), PF ratio (1.8%), leukocyte count (1.8%), platelet count (1.8%), creatinine (1.8%), temperature (1.2%), PEEP (0.6%), hematocrit (0.6%), sodium (0.6%), and bilirubin (0.6%). MICE generated multiple complete datasets where each missing value is estimated through an iterative series of predictive models based on the other variables in the dataset. Distribution of parent and imputed datasets is outlined in **Supplemental Figures 2** and **3** (https://links.lww.com/CCX/B544). Clustering analyses were performed on a final dataset that accounted for within-imputation and between-imputation uncertainty. As a sensitivity analysis, class assignment was performed separately on each imputed dataset with greater than 95% stability in class assignment.

Kaplan-Meier curves were generated using the *survfit* function from the survival package in R. Day 0 was defined as day of intubation and patients were followed for up to 90 days or censoring (death). Survival analysis was performed using Kaplan-Meier survival curves with log-rank tests for comparison between classes. Cox proportional hazards modeling was used to estimate hazard ratios (HRs) and 95% CIs for mortality risk between classes. The proportional hazards assumption was assessed graphically and found to be satisfied. Hospital-free days and ventilator-free days were calculated as previously described in critical care literature. Hospital-free days at 28 and 90 days were calculated as the number of days alive and outside the hospital during the respective time periods, with patients who died assigned a value of zero. Ventilator-free days were calculated as the number of days alive and free from mechanical ventilation within 28 days of ARDS onset, with patients who died or remained on mechanical ventilation for the entire period assigned a value of zero. There was no loss to follow-up in this 90-day window.

## RESULTS

### Cohort Description

The cohort included 166 adult patients who underwent HCT at Mayo Clinic Rochester (2005–2021), Florida or Arizona (2019–2021) and developed ARDS within 120 days of HCT (3, 5, 28). Of these, 96 (57.8%) received allogeneic and 70 (42.2%) autologous HCT. A study flow diagram is presented in **Supplemental Figure 4** (https://links.lww.com/CCX/B544). The median patient age at HCT was 58 (IQR: 51–65) years, with 54.8% being male. Most participants were White (85.5%) and non-Hispanic (74.7%). Baseline cohort characteristics are outlined in **Supplemental Table 2** (https://links.lww.com/CCX/B544).

### Latent Class Model Selection

**Figure 1.**
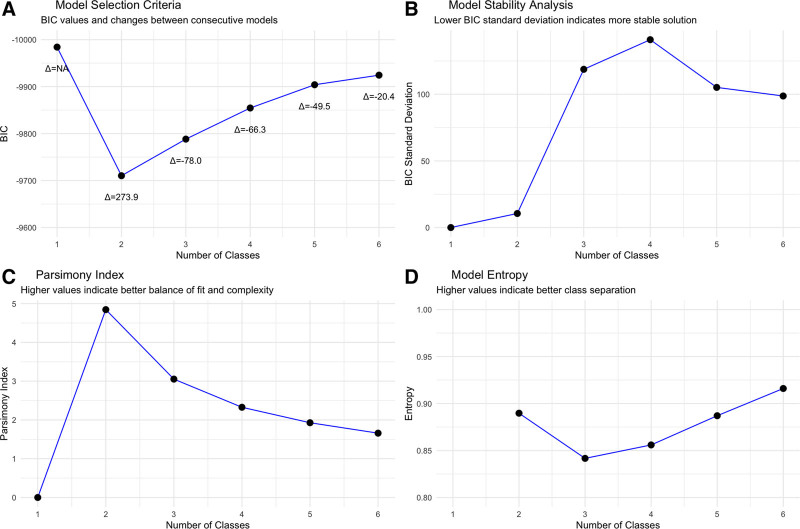
Model selection metrics for latent class analysis. **A**, Bayesian Information Criteria (BIC) values for models with one to six classes. Delta values show changes between consecutive models, with the largest improvement occurring between 1- and 2-class solutions. **B**, Model stability analysis showing BIC sd across bootstrap replicates. Lower values indicate more stable solutions, with the two-class model demonstrating good stability. **C**, Parsimony index, which balances model fit and complexity, showing optimal value for the two-class solution. **D**, Model entropy, reflecting class separation quality, with higher values indicating better class distinction. Together, these metrics support a two-class solution as optimal for this dataset.

The cohort was analyzed for between one and six latent classes with key metrics including BIC, stability of BIC, parsimony index, smallest class size, model entropy and VLMR (**Fig. 1** and **Table 1**; **Supplemental Fig. 5**, https://links.lww.com/CCX/B544). A two-class solution represented the best fit for the data available across multiple metrics. Specifically, this represented the largest improvement in BIC as well as lowest BIC (2.73% improvement from one class) with decline in BIC after two classes. The two-class solution had VLMR superiority over a one-class solution and further classes did not lead to an improvement in fit. The two-class solution had high entropy (0.9), the highest stability and best parsimony index. There were 81 patients assigned to class 1 and 85 patients assigned to class 2.

**TABLE 1.** Latent Class Analysis Model Characteristics

Model Classes	1	2	3	4	5	6
Class 1 size	166	81	38	73	44	37
Class 2 size	N/A	85	80	30	35	26
Class 3 size	N/A	N/A	40	25	10	10
Class 4 size	N/A	N/A	N/A	38	36	36
Class 5 size	N/A	N/A	N/A	N/A	41	9
Class 6 size	N/A	N/A	N/A	N/A	N/A	48
BIC	9984.24	9712.11	9915.79	9961.85	9971.93	10,000.54
BIC % change	N/A	2.73	–2.10	–0.47	–0.10	–0.29
BIC sd	0	10.54	118.75	140.96	105.17	98.72
Entropy	0	0.881	0.778	0.848	0.855	0.875
Parsimony index	0	0.048	0.028	0.023	0.019	0.016
VLMR test	N/A	0.001	0.776	0.549	0.417	0.200

BIC = Bayesian Information Criteria, N/A = not applicable, VLMR = Vuong-Lo-Mendell-Rubin Likelihood ratio.

A two-class solution was the optimal solution across multiple metrics including lowest BIC, model stability (lowest model BIC), best entropy, best parsimony index and significant VLMR *p* value.

### Latent Class Model Characteristics

**Figure 2.**
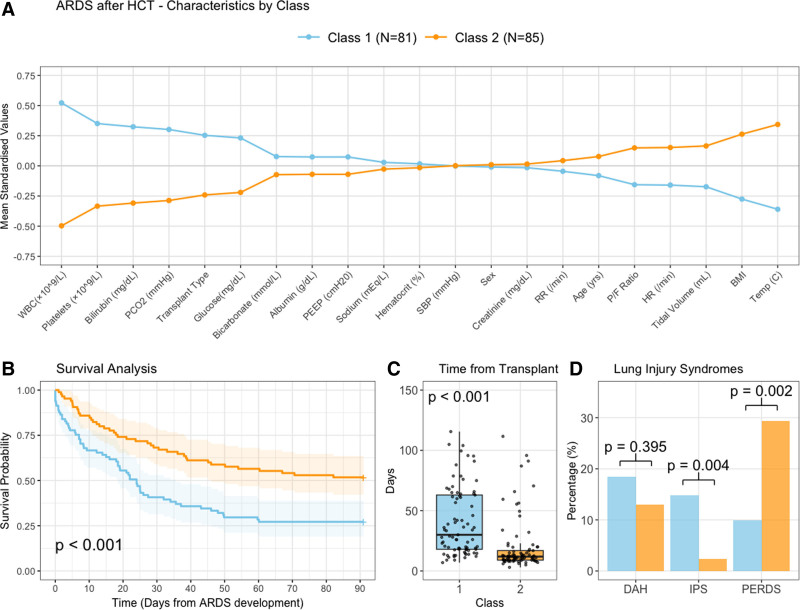
Class characteristics and clinical outcomes in post-hematopoietic acute respiratory distress syndrome. **A**, Mean standardized values of clustering variables by class. Variables are ordered by magnitude of difference between classes. Class 1 (*N* = 81, blue) shows higher WBC and platelet counts, higher bilirubin and Pco_2_, but lower temperature compared with class 2 (*N* = 85, orange). **B**, Kaplan-Meier survival curves demonstrating significantly worse 90-day survival in class 1 compared with class 2 (*p* < 0.001). Shaded areas represent 95% CIs. **C**, Time from transplant to ARDS onset by class (box‑and‑jitter plot); class 1 presented later after transplant than class 2 (*p* < 0.001). **D**, Distribution of noninfectious lung injury syndromes between classes. Class 2 had significantly higher rates of peri-engraftment respiratory distress syndrome (PERDS, *p* = 0.002), although class 1 had higher rates of idiopathic pneumonia syndrome (IPS, *p* = 0.004). No significant difference was observed in diffuse alveolar hemorrhage (DAH, *p* = 0.395).

The characteristics of the two classes are outlined in **Figure 2*A***, organized by mean standardized differences between classes (**Supplemental Table 3**, https://links.lww.com/CCX/B544). Class-defining, pre-transplant and diagnosis characteristics between classes are outlined in **Tables 2** and **3**. Boxplots outlining all class-defining and pre-transplant variables by assigned class are outlined in **Supplemental Figures 6** and **7** (https://links.lww.com/CCX/B544).

**TABLE 2.** Class-Defining Variables by Class

Characteristic	Class 1 (*n* = 81)	Class 2 (*n* = 85)	*p*
Class-defining variables			
Age (yr)	58.5 (47.7, 63.9)	58.1 (51.3, 64.8)	0.730
Body mass index	26.5 (23.8, 29.5)	28.6 (25.5, 33.0)	< 0.001
Pao_2_/Fio_2_ ratio	156.4 (95.4, 232.7)	211.0 (146.0, 278.1)	0.002
Pco_2_ (mm Hg)	41.0 (35.0, 51.0)	36.0 (31.0, 43.0)	< 0.001
Tidal volume (mL)	413.5 (114.5)	472.0 (223.5)	0.038
Positive-end expiratory pressure (cm H_2_O)	8.6 (3.1)	8.2 (3.8)	0.405
Temperature (°C)	37.5 (0.85)	38.2 (1.11)	< 0.001
Heart rate (/min)	119.5 (23.7)	126.4 (20.0)	0.044
Respiratory rate (/min)	33.9 (6.7)	34.5 (6.5)	0.572
Systolic blood pressure (mm Hg)	80.6 (24.6)	80.6 (13.2)	0.986
Hematocrit (%)	24.4 (21.9, 27.2)	25.1 (21.9, 27.0)	0.948
WBC (×10^9^/L)	5.9 (1.2, 12.4)	0.4 (0.2, 2.2)	< 0.001
Platelets (×10^9^/L)	27.0 (13.0, 55.0)	16.0 (10.0, 23.2)	< 0.001
Creatinine (mg/dL)	1.5 (1.1, 2.2)	1.5 (1.1, 2.4)	0.801
Bicarbonate (mmol/L)	18.2 (14.5, 25.0)	18.0 (16.0, 22.0)	0.813
Sodium (mEq/L)	142.4 (6.2)	141.9 (5.7)	0.606
Glucose (mg/dL)	194.0 (166.0, 231.0)	162.0 (138.8, 197.5)	0.001
Albumin (g/dL)	2.7 (2.3, 3.1)	2.7 (2.2, 3.1)	0.680
Bilirubin (mg/dL)	1.4 (0.6, 4.0)	0.9 (0.5, 1.4)	< 0.001
Transplant type			0.001
Autologous	24 (29.6%)	46 (54.1%)	
Allogeneic	57 (70.4%)	39 (45.9%)	
Sex			0.900
Female	37 (45.7%)	38 (44.7%)	
Male	44 (54.3%)	47 (55.3%)	

Numbers indicate *n* (%), mean (sd), or median (interquartile range).

**TABLE 3.** Pre-transplant Variables and Outcomes by Class

Characteristic	Class 1 (*n* = 81)	Class 2 (*n* = 85)	*p*
Pre-transplant variables			
WBC (×10^9^/L)	1.7 (0.4, 3.4)	2.1 (0.6, 4.7)	0.029
Platelets (×10^9^/L)	41.0 (10.0, 109.8)	75.0 (38.0, 119.0)	0.023
Hemoglobin (g/dL)	7.8 (7.1, 9.2)	8.7 (7.6, 10.3)	0.004
Lactate dehydrogenase (IU/L)	318.0 (231.0, 474.0)	252.0 (201.0, 322.0)	0.006
Bicarbonate (mmol/L)	22.0 (20.0, 24.0)	22.0 (20.0, 26.0)	0.122
Creatinine (mg/dL)	1.0 (0.9, 1.3)	1.0 (0.8, 1.4)	0.842
Albumin (g/dL)	3.2 (2.9, 3.6)	3.3 (2.9, 3.5)	0.509
Aspartate aminotransferase (U/L)	46.0 (29.0, 100.0)	36.0 (26.0, 58.0)	0.014
Left ventricular ejection fraction (%)	59.0 (8.0)	60.4 (6.6)	0.220
Right ventricular systolic pressure (%)	29.8 (8.2)	30.8 (7.2)	0.404
Forced expiratory volume in 1 sec *z* score	–0.84 (1.08)	–0.73 (1.14)	0.522
Forced vital capacity *z* score	–0.71 (1.11)	–0.72 (1.10)	0.962
Diffusing capacity of the lungs for carbon monoxide *z* score	–2.52 (1.39)	–2.11 (1.28)	0.056
Endothelial Activation and Stress Index	1.5 (0.8, 5.5)	1.05 (0.6, 2.55)	0.001
Time-to-acute respiratory distress syndrome (d)	30.0 (18.0, 63.0)	11.9 (9.0, 16.9)	< 0.001
Outcomes			
Hospital-free days (28 d)	3.19 (7.16)	5.36 (8.28)	0.074
Hospital-free days (90 d)	5.38 (12.21)	12.99 (17.64)	0.002
Mortality (28 d)	48 (59.3%)	25 (29.4%)	< 0.001
Mortality (90 d)	59 (72.8%)	41 (48.2%)	0.001
In-hospital mortality	52 (64.2%)	36 (42.4%)	0.005
Peri-engraftment respiratory distress syndrome	8 (9.9%)	25 (29.4%)	0.002
Idiopathic pneumonia syndrome	12 (14.8%)	2 (2.4%)	0.004
Diffuse alveolar hemorrhage	15 (18.5%)	11 (12.9%)	0.326

Numbers indicate *n* (%), mean (sd), or median (interquartile range). Endothelial Activation and Stress Index calculated as [creatinine × lactate dehydrogenase]/platelets) pre-stem cell infusion.

Although transplant type (autologous vs. allogeneic) was included as a class-defining variable, both classes contained a mix of transplant types. Class 1 had a higher proportion of allogeneic transplants (70.4% vs. 45.9%, *p* = 0.001), but still included a substantial number of autologous recipients. Similarly, class 2 contained both allogeneic (45.9%) and autologous (54.1%) recipients. Those assigned to class 1 had higher pre-transplant EASIX (8.1 vs. 4.1, *p* = 0.002) and lower pre-transplant DLCO *z* score (–2.4 vs. –2.0, *p* = 0.04) compared with class 2. There were no differences between classes for pre-transplant smoking status, renal, cardiac function or spirometry. In terms of pre-ARDS critical care syndromes, there were no differences in the frequency of pneumonia, bloodstream infection or septic shock between the two classes.

At the time of ARDS development, those in class 1 were more likely to have lower BMI (26.5 vs. 28.6 kg/m^2^, *p* < 0.001), have worse hypoxemia (P/F ratio 156.4 vs. 211.0, *p* = 0.005), and higher Pco_2_ (41.0 vs. 36.0 kPa, *p* < 0.001) when compared with patients in class 2. Class 1 patients were also less likely to have features suggestive of an inflammatory response including temperature (37.5°C vs. 38.2°C, *p* < 0.001). Blood counts were higher in class 1, consistent with the later time-to-ARDS development. Specifically, class 1 had higher WBC (5.9 vs. 0.4 × 10^9^/L, *p* < 0.001) and platelet (27.0 vs. 16.0 × 10^9^/L, *p* < 0.001) counts. Other notable differences between classes included higher glucose (194.0 vs. 162.0 mg/dL, *p* = 0.001) and bilirubin (1.4 vs. 0.9 mg/dL, *p* < 0.001) in class 1. Classification stability was modest (0.623 ± 0.346) across 1000 Monte Carlo simulations.

### Patient Outcomes Stratified by Class

Patient outcomes by class are outlined in Table 3 with survival analyses in **Figure 2*B***. Those in class 1 had a longer time from HCT to ARDS development compared with patients in class 2 (30.0 vs. 11.9 d, *p* < 0.001; **Fig. 2*C***). Unadjusted outcomes were significantly worse for class 1. Thus, those who were assigned to class 1 had worse overall survival (HR: 0.48; 95% CI, 0.32–0.72; *p* < 0.001), higher 28-day (59.3% vs. 29.4%, *p* < 0.001) and 90-day mortality (72.8% vs. 48.2%, *p* = 0.001) and fewer hospital-free days at 90 days (0.0 [IQR: 0.0–1.1] vs. 0.0 [IQR: 0.0–22.8], *p* < 0.001) vs. patients in class 2. Total duration of IMV was similar between groups (class 1: 6.2 [IQR: 2.7–11.9], class 2: 7.0 d [IQR: 3.0–14.8] , *p* = 0.36). Ventilator-free days was lower in class 1 (0.0 [IQR: 0.0–21.6] vs. 15.8 d [IQR: 0.0–23.5], *p* = 0.009). Diagnosis of HCT-specific lung injury syndromes are outlined in **Figure 2*D***. PERDS was more likely in class 2 (25/85, 29.4% vs. 8/81, 9.9%, *p* = 0.002) and IPS was more likely in class 1 (12/85, 14.8% vs. 2/81, 2.4%, *p* = 0.004). Frequency of DAH did not differ between classes (class 1: 15/85, 18.5%, class 2: 11/81, 12.9%; *p* = 0.40). These patterns were consistent in pre-specified subgroup analyses separating allogeneic and autologous HCT (**Supplemental Figs 8**–**10**, https://links.lww.com/CCX/B544). Analysis of outcomes by specific lung injury syndromes revealed no statistically significant mortality differences between classes within individual syndromes.

### Parsimonious Model Development

**Figure 3.**
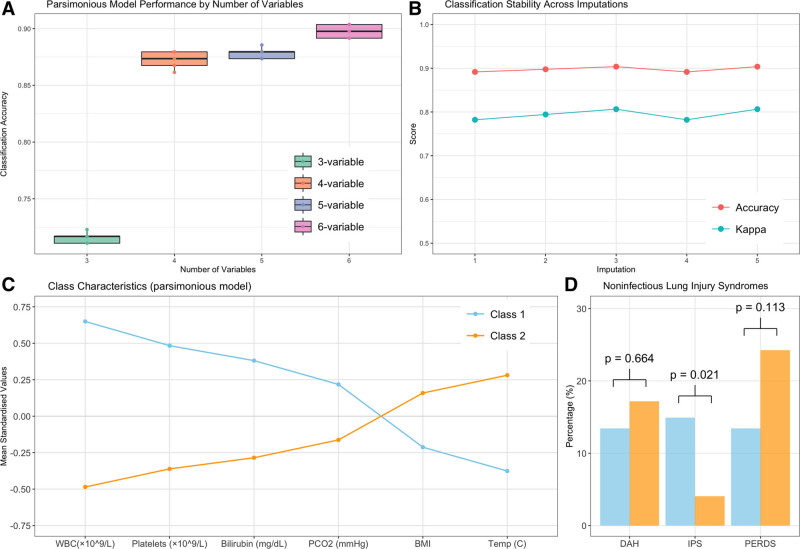
Development and validation of parsimonious model. **A**, Classification accuracy improves with increasing number of variables in the model, reaching optimal performance with six variables. *Box plots* show the distribution of accuracy scores across validation sets. **B**, Model stability assessment across five multiple imputations showing consistently high accuracy (*red*) and kappa statistics (*blue*), indicating robust classification performance regardless of imputation. **C**, Class characteristics using the parsimonious six-variable model. Mean standardized values show similar patterns to the full model, with WBC and platelet counts being the strongest discriminators between class 1 (*blue*) and class 2 (*orange*). **D**, Distribution of noninfectious lung injury syndromes using the parsimonious model classification. Although idiopathic pneumonia syndrome (IPS) remains significantly different between classes (*p* = 0.021), differences in peri-engraftment respiratory distress syndrome (PERDS) become less pronounced (*p* = 0.113) compared with the full model. Diffuse alveolar hemorrhage (DAH) shows no significant difference between classes (*p* = 0.664).

As an exploratory analysis, we developed a parsimonious model to replicate class assignments using a minimal set of key variables (Fig. 2*A*; and Supplemental Table 3, https://links.lww.com/CCX/B544). Classification accuracy was modest with a three-variable model but improved to 0.90 with a six-variable model (**Fig. 3*A***) and remained stable across imputations (**Fig. 3*B***). Variables chosen were WBC count, platelet count, bilirubin, Pco_2_, BMI, and temperature (**Fig. 3*C***). The parsimonious model’s ability to discriminate IPS remained but performance for identifying PERDS decreased (**Fig. 3*D***).

## DISCUSSION

This multicenter study of post-HCT ARDS used data available at ARDS onset to identify two distinct phenotypes with different clinical characteristics and outcomes. Although class 1 had more allogeneic transplants and class 2 had more autologous transplants, both classes included substantial numbers of each transplant type. This suggests the phenotypes capture biological processes that transcend a simple autologous/allogeneic dichotomy. Class 1 exhibited more severe respiratory physiology (worse hypoxemia, higher Pco_2_) and evidence of multi-organ dysfunction (particularly elevated bilirubin). Without time being incorporated as a classification variable, class 1 nevertheless emerged as a later-onset phenotype occurring around day 30 post-transplant, with significantly higher prevalence of IPS and substantially worse 90-day mortality (72.8%). In contrast, class 2 manifested during the peri-engraftment period and was distinguished by profound cytopenia, fever, and tachycardia. This phenotype showed a significantly higher prevalence of PERDS. Despite substantial burden of critical illness and severe neutropenia at the time of ARDS, those in class 2 had better survival.

These findings extend prior ARDS phenotyping work in several important ways. Although previous studies have identified hyperinflammatory and hypoinflammatory ARDS phenotypes in general critical care populations (20–22), our results suggest that post-HCT ARDS may follow different patterns of heterogeneity more closely tied to the degree of immune reconstitution at the time of ARDS development (2, 7, 16, 27). This aligns with the unique pathophysiology of post-HCT lung injury, where conditioning-induced cytopenias, neutrophil recovery, engraftment, and graft-vs.-host disease create distinct inflammatory environments at different time points (2, 36, 61, 62). The cytopenic ARDS phenotype coincides with neutrophil recovery, whereas the post-engraftment ARDS phenotype may reflect the emergence of alloreactivity and evolving chronic complications (4, 9, 12, 27, 28, 62–64). Notably, these phenotypes share parallels with established ARDS phenotypes from general critical care populations. Class 2 demonstrated features that may be associated with inflammatory response patterns, including higher fever (38.2°C vs. 37.5°C, *p* < 0.001) and occurred during the peri-engraftment period when inflammatory processes are typically heightened. Although both classes exhibited tachycardia consistent with critical illness, class 1 was characterized by more severe respiratory failure and higher mortality, patterns that have been observed in hypoinflammatory ARDS phenotypes in other populations.

The prominence of bilirubin as a discriminating variable aligns with its established role in ARDS phenotyping studies, although our findings suggest context-specific interpretation may be necessary. Unlike general ARDS populations where elevated bilirubin typically indicates hyperinflammatory phenotype, the elevated bilirubin in our class 1 patients may reflect HCT-specific hepatic complications including conditioning toxicity, medication effects, or graft-vs.-host disease.

We also developed a parsimonious model using just six readily available clinical variables (leukocyte count, platelet count, bilirubin, Pco_2_, BMI, and temperature) that reproduced full-model phenotype assignments with 90% accuracy. Although likely overfitted, this simplified model could support real-time classification in future studies without complex computation. Notably, although this reduced model maintained good discrimination for IPS between classes, its ability to identify PERDS decreased, suggesting that additional variables in the full model may be important for capturing specific post-HCT lung injury patterns.

Several potentially important variables were not included in our analysis. Dynamic lung compliance and lung injury scores, whereas clinically relevant, were not consistently documented across our study period and sites, limiting their practical availability. Similarly, precise fluid balance data, transfusion requirements, and detailed pre-transplant therapy exposure represent important variables that could refine phenotype identification but were not consistently available in our retrospective cohort. Future prospective studies should consider incorporating these variables alongside standardized data collection protocols to capture more comprehensive physiologic and treatment-related information that may improve phenotype discrimination and clinical utility.

Our analysis was limited to variables available at the time of ARDS diagnosis, but longitudinal respiratory physiology data over the first 24–72 hours could significantly strengthen contextualization of observed phenotypes. Trajectory patterns of oxygenation, ventilator requirements, and organ dysfunction scores could provide important validation of initial phenotype assignments and potentially identify patients whose classification might evolve with additional clinical information. Such longitudinal approaches could also determine whether early phenotype classification remains stable or whether incorporating physiologic trajectories improves prognostic accuracy. This represents an important area for future prospective validation studies with standardized serial data collection protocols.

This study has several important strengths. First, the cohort size is notable given that post-HCT ARDS occurs in only 5% of transplant recipients and large-scale studies are lacking. Second, our analytical approach was statistically rigorous, using multiple complementary metrics—BIC, model stability, entropy, and VLMR ratios—to validate the phenotyping strategy. The consistent two-class solution across different validation approaches strengthens confidence in our findings. Third, our model used only variables available at ICU admission, enhancing its potential clinical utility. Fourth, comprehensive data collection, with low rates of missing data enabled robust multiple imputation and sensitivity analyses. Fifth, by including both autologous and allogeneic transplant recipients, we identified phenotypes that transcend this traditional categorization, reflecting the clinical reality where physicians must manage ARDS in both populations. Finally, this study represents the first systematic attempt to understand heterogeneity in post-HCT ARDS using LCA, providing novel insights into this challenging clinical syndrome.

This study also has several limitations. First, although our cohort represents one of the largest studies of post-HCT ARDS, the sample size (*n* = 166) remains below the recommended minimum of 300 cases for optimal LCA development. However, post-HCT ARDS occurs in only 5% of transplant recipients, making large-scale studies logistically challenging, and our cohort represents by far the largest ARDS cohort after HCT studied to date. Consistent with this limitation, Monte Carlo simulations showed modest reproducibility (0.623 ± 0.346), and the absence of a traditional development-validation split due to sample size constraints affects the generalizability of our findings. External validation in independent cohorts remains essential before these phenotypes can be considered clinically actionable. Second, our study spans a 16-year period during which both hematologic and critical care practices evolved substantially, with changes in conditioning regimens, prophylaxis strategies, and ICU management protocols that may have influenced both phenotype development and outcomes in ways not captured by our analysis. Future validation studies should focus on more contemporary cohorts with standardized protocols and larger sample sizes to ensure optimal statistical power and contemporary clinical relevance. Third, and perhaps most crucially, the observational nature of this study meant we lacked biological samples to correlate these clinical phenotypes with underlying molecular mechanisms. Although our analysis suggests that certain pathophysiological processes may be common to both transplant types, biological validation is needed to confirm these patterns. Fourth, our findings from a single healthcare system, albeit across multiple centers, may not fully generalize to other transplant populations. Fifth, our survival and outcome analyses were not adjusted for potential confounders through multivariable modeling. Although the LCA approach inherently accounts for multiple variables simultaneously in defining phenotypes, the subsequent outcome comparisons represent unadjusted analyses. This was intentional as our primary goal was to describe the natural history and outcomes of the identified phenotypes rather than establish causal relationships. However, this limitation warrants caution in interpreting the prognostic implications of these phenotypes, and future studies should incorporate multivariable adjustment to control for potential confounding variables such as disease severity, comorbidities, and treatment variations.

## CONCLUSIONS

This study represents an important step forward in understanding post-HCT ARDS heterogeneity. The identification of timing-based phenotypes with distinct clinical characteristics and outcomes provides a framework for future research and could inform clinical care. Prospective validation of these phenotypes, ideally with biological correlates, represents an important next step in improving outcomes for this high-risk patient population.

## ACKNOWLEDGMENTS

We acknowledge Anesthesia Clinical Research Unit Data Specialist Ms. Danette Bruns, R.R.T., L.R.T. for their help with data extraction.

## Supplementary Material
