# Supplementary material for: Acute Respiratory Distress Syndrome Phenotypes After Stem Cell Transplantation: A Latent Class Analysis
Source: Crit Care Explor. 2025 Sep 5;7(9):e1312. doi: 10.1097/CCE.0000000000001312 (PMC12417010; doi:10.1097/CCE.0000000000001312)
Supplement: Supplementary file 1 [file cc9-7-e1312-s001.pdf]

Acute Respiratory Distress Syndrome Phenotypes After Stem Cell Transplantation: A Latent Class Analysis

Electronic Data Supplement – Supplemental Tables

1. **Supplemental Table 1. Strengthening the Reporting of Observational Studies in Epidemiology (STROBE) checklist.**

2. **Supplemental Table 2. Baseline Patient Demographics and Clinical Data**

3. **Supplemental Table 3. Mean Differences Between Class 1 and Class 2**

Electronic Data Supplement – Supplemental Figures

1. **Supplemental Figure 1. Correlation Matrix of Candidate Clustering Variables.** Heatmap showing pairwise Pearson correlation coefficients between clinical variables considered for latent class analysis. Color intensity represents correlation strength, with red indicating positive correlations and blue indicating negative correlations (scale from -1 to +1). Variables include vital signs (SBP, HR, RR, Temp), laboratory values (albumin, bicarbonate, bilirubin, creatinine, hematocrit, WBC, platelets, sodium), ventilator parameters (tidal volume, minute ventilation, PEEP), and gas exchange measures (P/F ratio, PCO2). Notable correlations include the relationship between pH and bicarbonate (r=0.58), and between tidal volume and minute ventilation (r=0.58), which led to the exclusion of pH and minute ventilation from the final clustering model to avoid collinearity.

2. **Supplemental Figure 2. Distribution comparison between original and imputed values using density plots.** Density plots comparing the distribution of original values (blue line) with five multiple imputations (colored lines) for each clinical variable used in the latent class analysis. Variables include ventilator parameters (P/F ratio, tidal volume, PEEP), vital signs (temperature), laboratory values (hematocrit, WBC, platelets, creatinine, bicarbonate, sodium, glucose, albumin, bilirubin). The overlapping distributions demonstrate the preservation of the original data structure across multiple imputations, supporting the validity of the imputation process.

3. **Supplemental Figure 3. Box plot comparison between original and imputed values.** Box plots comparing the distribution of original values (blue) with five multiple imputations (colored) for each clinical variable used in the latent class analysis. The boxes show the interquartile range (IQR) with median line, whiskers extend to 1.5×IQR, and individual points represent outliers. Variables are the same as in Supplemental Figure 2. The consistent median values and similar spread across imputations demonstrate the robustness of the multiple imputation approach in maintaining the underlying data structure while accounting for missing values.

4. **Supplemental Figure 4. Study Flow Diagram.**

5. **Supplemental Figure 5. Model Selection Metrics for Latent Class Analysis.** (A) Size of smallest class as a percentage of total cohort. The dashed line at 25% represents a threshold for potentially unstable small class sizes. (B) Relative BIC improvement shown as percentage change from previous model, demonstrating largest improvement with the 2-class solution.

6. **Supplemental Figure 6. Class-Defining Variables.** Box plots comparing the distribution of clinical variables at time of ARDS diagnosis between Class 1 (blue) and Class 2 (orange). Variables include demographics (age, BMI), vital signs (temperature, heart rate, respiratory rate, systolic blood pressure), laboratory values (albumin, bicarbonate, bilirubin, creatinine, glucose, hematocrit, platelets, sodium, white blood cell count), and respiratory parameters (P/F ratio, PCO2, PEEP, tidal volume). The boxes show interquartile range with median line, whiskers extend to 1.5×IQR, and points represent outliers. Notable differences include lower P/F ratios, higher PCO2, and higher WBC counts in Class 1 compared to Class 2.

7. **Supplemental Figure 7. Pre-Transplant Variables by Class.** Box plots comparing the distribution of pre-transplant clinical variables between Class 1 (blue) and Class 2 (orange). Variables include laboratory values (albumin, AST, bicarbonate, creatinine, hemoglobin, LDH, platelets, WBC), cardiac function (LVEF), pulmonary function tests (DLCO Z-score, FEV1 Z-score, FVC Z-score, RVSP), and risk scores (EASIX). Notable differences include lower DLCO Z-scores and higher EASIX scores in Class 1, suggesting greater pre-transplant organ dysfunction in this group.

8. **Supplemental Figure 8. Sensitivity Analysis: Survival in Allogeneic Transplant Recipients.** Kaplan-Meier survival curves comparing Class 1 (blue) and Class 2 (orange) among allogeneic HCT recipients who developed ARDS. Shaded areas represent 95% confidence intervals. The number of patients at risk is shown below the graph at 10-day intervals. While Class 1 showed numerically worse survival, the difference did not reach statistical significance in the allogeneic subgroup ($p = 0.155$).

9. **Supplemental Figure 9. Sensitivity Analysis: Survival in Autologous Transplant Recipients.** Kaplan-Meier survival curves comparing Class 1 (blue) and Class 2 (orange) among autologous HCT recipients who developed ARDS. Shaded areas represent 95% confidence intervals. The number of patients at risk is shown below the graph at 10-day intervals. Class 1 demonstrated significantly worse survival compared to Class 2 in the autologous subgroup ($p = 0.005$).

10. **Supplemental Figure 10. Distribution of lung injury syndromes by transplant type and ARDS phenotype.** Bar plots showing the frequency of specific lung injury syndromes (DAH: diffuse alveolar hemorrhage, IPS: idiopathic pneumonia syndrome, PERDS: peri-engraftment respiratory distress syndrome) in Class 1 (blue) and Class 2 (orange), stratified by transplant type. Panel A shows allogeneic HCT recipients, while Panel B shows autologous HCT recipients. P-values for between-class comparisons are shown above each pair of bars. PERDS was significantly more common in Class 2 among autologous recipients ($p = 0.005$).

Supplemental Table 1. Strengthening the Reporting of Observational Studies in Epidemiology (STROBE) checklist.

	Item No	Recommendation	Page number
**Title and abstract**	1	(*a*) Indicate the study's design with a commonly used term in the title or the abstract	1
		(*b*) Provide in the abstract an informative and balanced summary of what was done and what was found	3-4
**Introduction**			
Background/rationale	2	Explain the scientific background and rationale for the investigation being reported	6
Objectives	3	State specific objectives, including any prespecified hypotheses	7
**Methods**			
Study design	4	Present key elements of study design early in the paper	7
Setting	5	Describe the setting, locations, and relevant dates, including periods of recruitment, exposure, follow-up, and data collection	7-8
Participants	6	(*a*) Give the eligibility criteria, and the sources and methods of selection of participants. Describe methods of follow-up	7-8
		(*b*) For matched studies, give matching criteria and number of exposed and unexposed	NA
Variables	7	Clearly define all outcomes, exposures, predictors, potential confounders, and effect modifiers. Give diagnostic criteria, if applicable	8-10
Data sources/ measurement	8*	For each variable of interest, give sources of data and details of methods of assessment (measurement). Describe comparability of assessment methods if there is more than one group	8-10
Bias	9	Describe any efforts to address potential sources of bias	7,8,10
Study size	10	Explain how the study size was arrived at	7
Quantitative variables	11	Explain how quantitative variables were handled in the analyses. If applicable, describe which groupings were chosen and why	9-10
Statistical methods	12	(*a*) Describe all statistical methods, including those used to control for confounding	8-11
		(*b*) Describe any methods used to examine subgroups and interactions	8-9, Suppl. Figure 1
		(*c*) Explain how missing data were addressed	10
		(*d*) If applicable, explain how loss to follow-up was addressed	NA
		(*e*) Describe any sensitivity analyses	11
**Results**			

Participants	13*	(a) Report numbers of individuals at each stage of study—eg numbers potentially eligible, examined for eligibility, confirmed eligible, included in the study, completing follow-up, and analysed	11
		(b) Give reasons for non-participation at each stage	NA
		(c) Consider use of a flow diagram	Suppl. Figure 4
Descriptive data	14*	(a) Give characteristics of study participants (eg demographic, clinical, social) and information on exposures and potential confounders	11, Suppl Table 2
		(b) Indicate number of participants with missing data for each variable of interest	Suppl Table 2
		(c) Summarise follow-up time (eg, average and total amount)	NA
Outcome data	15*	Report numbers of outcome events or summary measures over time	12
Main results	16	(*a*) Give unadjusted estimates and, if applicable, confounder-adjusted estimates and their precision (eg, 95% confidence interval). Make clear which confounders were adjusted for and why they were included	11-12
		(*b*) Report category boundaries when continuous variables were categorized	11
		(*c*) If relevant, consider translating estimates of relative risk into absolute risk for a meaningful time period	NA
Other analyses	17	Report other analyses done—eg analyses of subgroups and interactions, and sensitivity analyses	12-14
**Discussion**			
Key results	18	Summarise key results with reference to study objectives	14-15
Limitations	19	Discuss limitations of the study, taking into account sources of potential bias or imprecision. Discuss both direction and magnitude of any potential bias	16-17
Interpretation	20	Give a cautious overall interpretation of results considering objectives, limitations, multiplicity of analyses, results from similar studies, and other relevant evidence	15-17
Generalisability	21	Discuss the generalisability (external validity) of the study results	16
**Other information**			
Funding	22	Give the source of funding and the role of the funders for the present study and, if applicable, for the original study on which the present article is based	1

Supplemental Table 2. Baseline Patient Demographics and Clinical Data	
Characteristic	Median (IQR) or N (%)
All patients (N = 166)	
**Type of Transplant**	
Allogeneic	96 (57.8)
Autologous	70 (42.2)
**Location**	
Rochester, MN	154 (92.8)
Jacksonville, FL	7 (4.2)
Scottsdale, AZ	5 (3.0)
**Age at HCT, years**	58.2 (50.7, 64.7)
**Male sex**	91 (54.8)
**Weight (kg)**	76.9 (65.5, 91.0)
**Body mass index**	27.4 (24.6, 31.6)
**Race**	
White	142 (85.5)
Black or African American	4 (2.4)
Asian	3 (1.8)
American Indian/Alaskan Native	1 (0.6)
Other/mixed	1 (0.6)
Unknown/not disclosed	15 (9,0)
**Ethnicity**	
Not Hispanic or Latino	124 (74.7)
Hispanic or Latino	3 (1.8)
Not disclosed/unknown	39 (23.5)
**Disease**	
Acute myeloid leukemia	37 (22.3)
Multiple Myeloma	52 (31.3)
Non-Hodgkin lymphoma	28 (16.9)
Myelodysplastic syndrome	18 (10.8)
Acute lymphocytic leukemia	13 (7.8)
Other	18 (10.8)

Abbreviations: IQR = interquartile range; HCT = hematopoietic stem cell transplantation.

Supplemental Table 3.

Mean Differences Between Class 1 and Class 2

Label	Class 1 Mean	Class 2 Mean	Absolute Difference
Leukocyte count, 10^9/L	0.522	-0.497	1.019
Temperature, °C	-0.360	0.343	0.703
Platelet count, 10^9/L	0.351	-0.334	0.685
Bilirubin, mg/dL	0.324	-0.309	0.633
PCO2, mmHg	0.301	-0.287	0.589
Body mass index	-0.276	0.263	0.538
Transplant Type	0.253	-0.241	0.494
Glucose, mg/dL	0.231	-0.220	0.452
Tidal volume, mL	-0.173	0.165	0.339
Heart rate, /min	-0.160	0.152	0.312
PaO2/FiO2 ratio	-0.156	0.149	0.305
Age, years	-0.081	0.077	0.159
Bicarbonate, mmol/L	0.077	-0.073	0.150
Albumin, g/dL	0.074	-0.070	0.144
PEEP, cm H2O	0.074	-0.070	0.144
Respiratory rate, /min	-0.045	0.043	0.088
Sodium, mEq/L	0.029	-0.027	0.056
Hematocrit, %	0.017	-0.016	0.032
Creatinine, mg/dL	-0.015	0.015	0.030
Sex	-0.010	0.010	0.019
Systolic blood pressure, mmHg	-0.001	0.001	0.003

Class characteristics were compared using standardized mean differences, calculated as the difference between class means divided by the pooled standard deviation. Variables were standardized (z-scored) prior to latent class analysis, and the reported class means represent the mean standardized values for each class across the clustering variables.

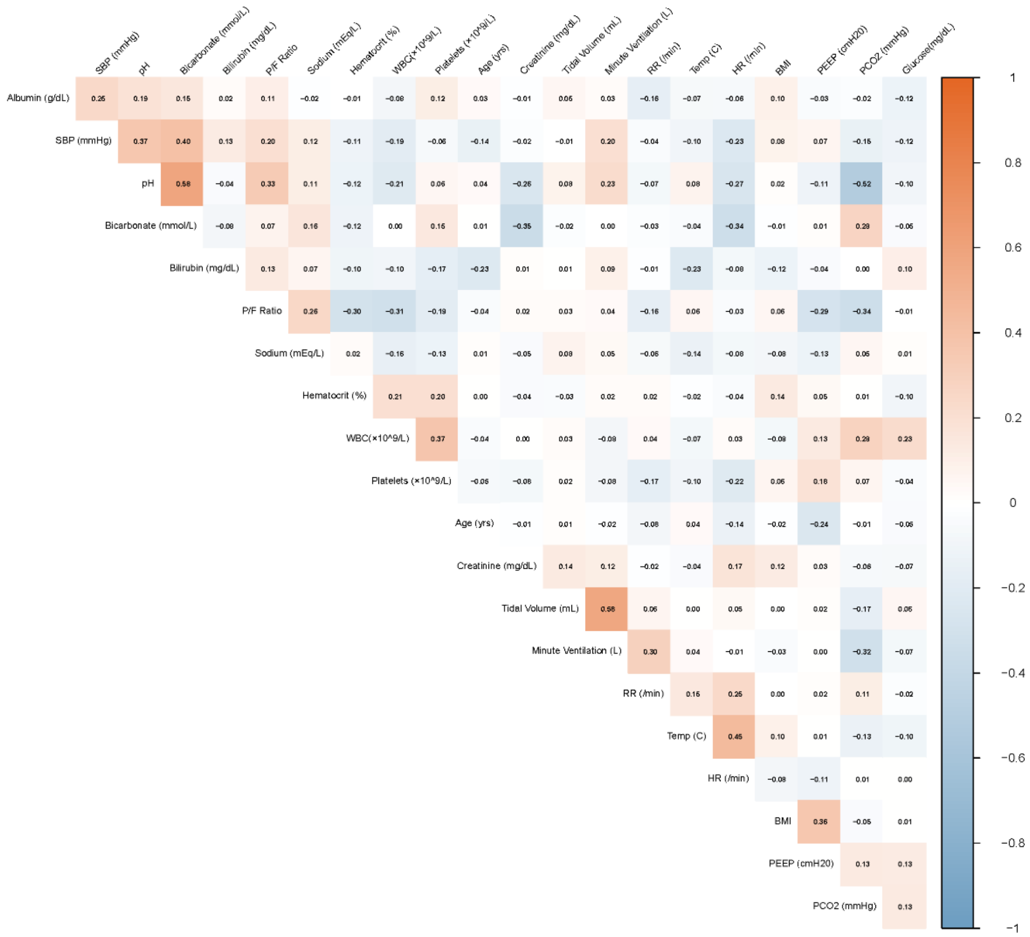

Supplemental Figure 1. Correlation Matrix of Candidate Clustering Variables. Heatmap showing pairwise Pearson correlation coefficients between clinical variables considered for latent class analysis. Color intensity represents correlation strength, with red indicating positive correlations and blue indicating negative correlations (scale from -1 to +1). Variables include vital signs (SBP, HR, RR, Temp), laboratory values (albumin, bicarbonate, bilirubin, creatinine, hematocrit, WBC, platelets, sodium), ventilator parameters (tidal volume, minute ventilation, PEEP), and gas exchange measures (P/F ratio, PCO2). Notable correlations include the relationship between pH and bicarbonate (r=0.58), and between tidal volume and minute ventilation (r=0.58), which led to the exclusion of pH and minute ventilation from the final clustering model to avoid collinearity.

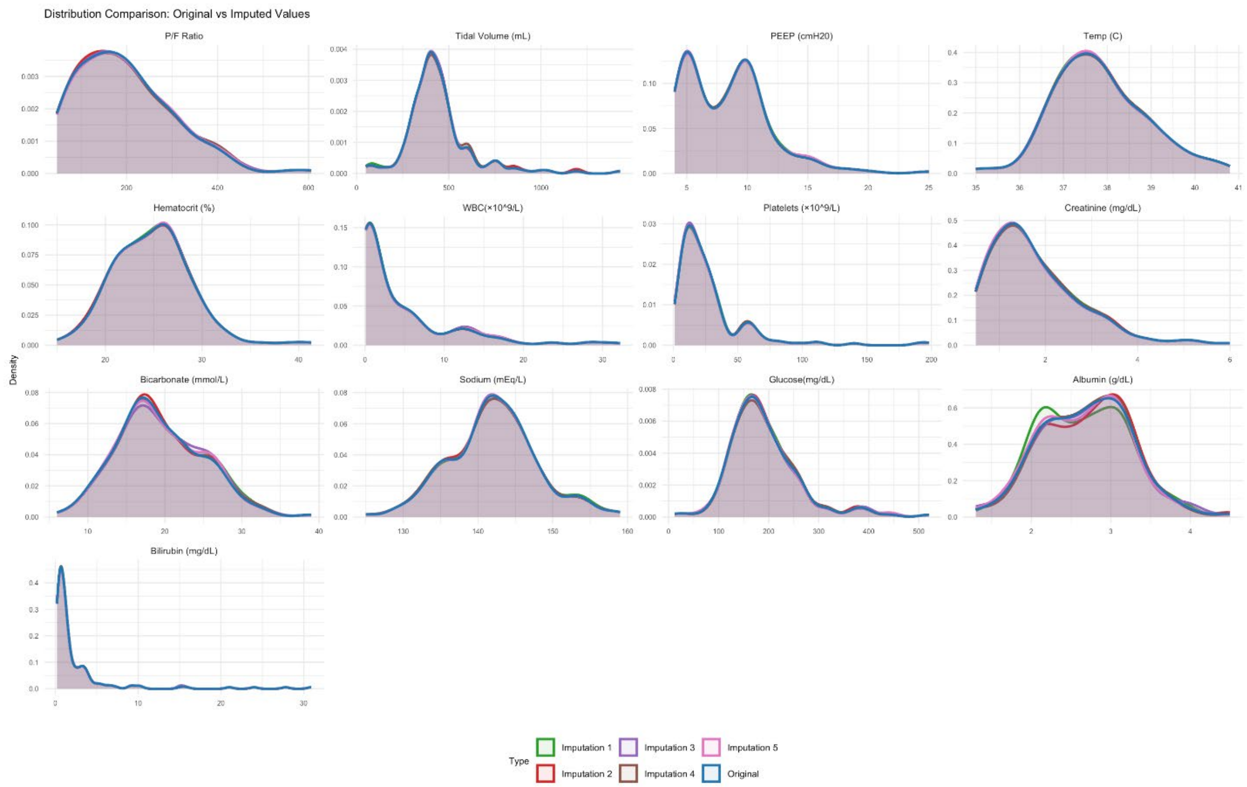

Supplemental Figure 2. Distribution comparison between original and imputed values using density plots. Density plots comparing the distribution of original values (blue line) with five multiple imputations (colored lines) for each clinical variable used in the latent class analysis. Variables include ventilator parameters (P/F ratio, tidal volume, PEEP), vital signs (temperature), laboratory values (hematocrit, WBC, platelets, creatinine, bicarbonate, sodium, glucose, albumin, bilirubin). The overlapping distributions demonstrate the preservation of the original data structure across multiple imputations, supporting the validity of the imputation process.

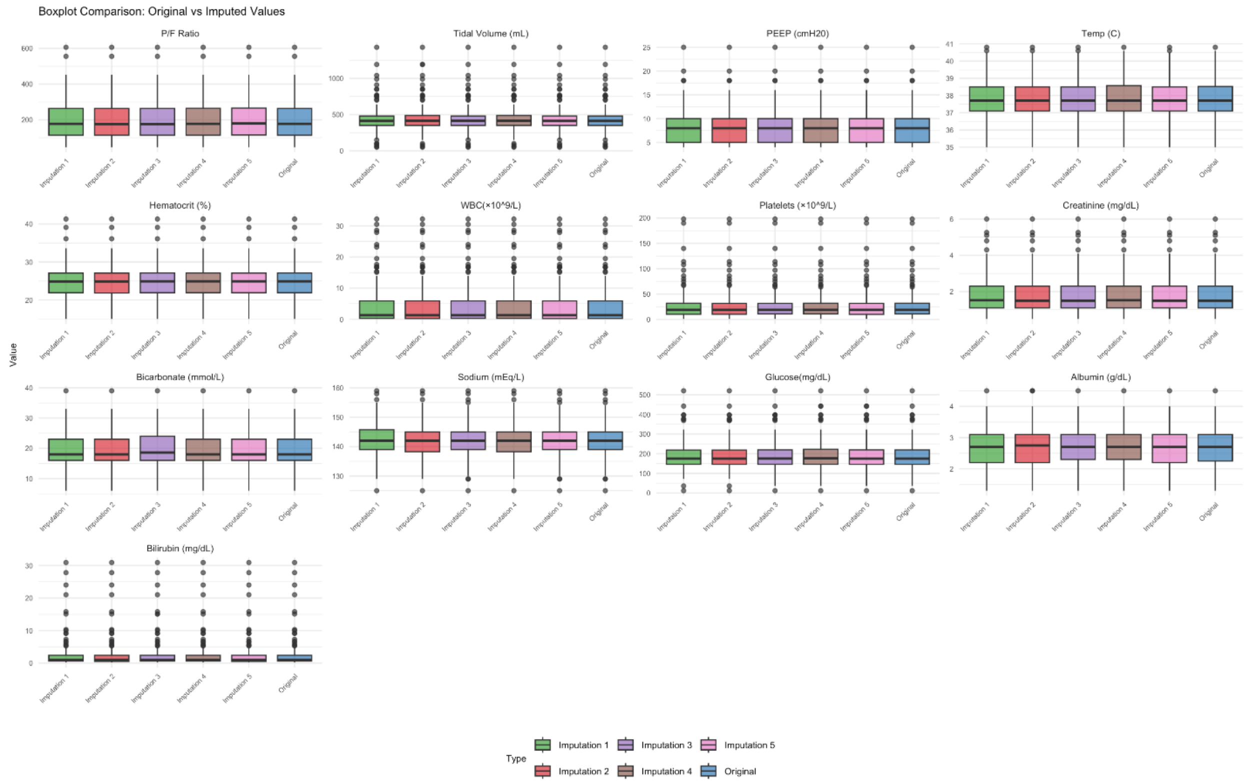

Supplemental Figure 3. Box plot comparison between original and imputed values. Box plots comparing the distribution of original values (blue) with five multiple imputations (colored) for each clinical variable used in the latent class analysis. The boxes show the interquartile range (IQR) with median line, whiskers extend to 1.5×IQR, and individual points represent outliers. Variables are the same as in Supplemental Figure 2. The consistent median values and similar spread across imputations demonstrate the robustness of the multiple imputation approach in maintaining the underlying data structure while accounting for missing values.

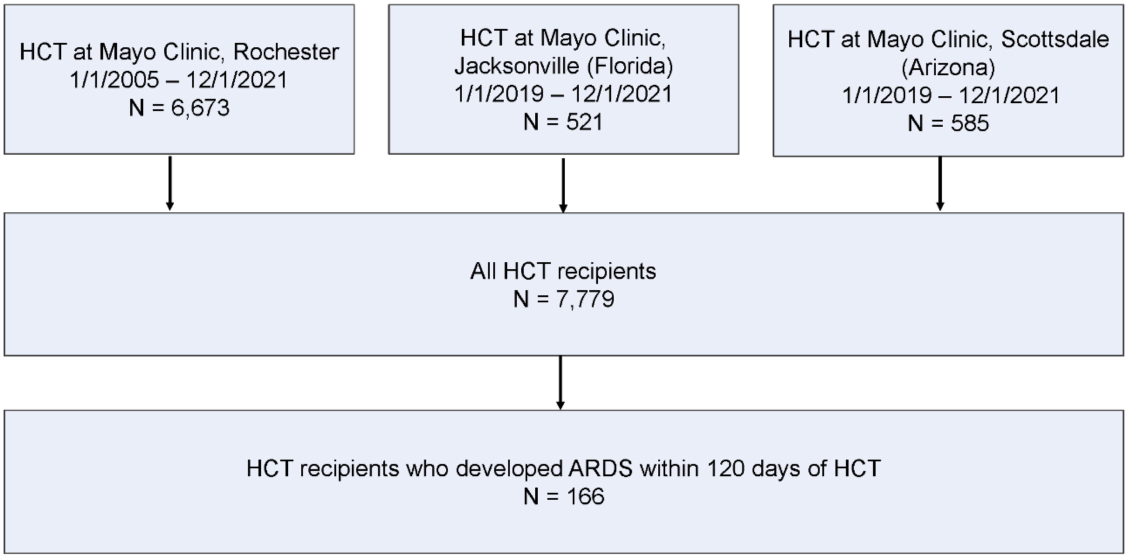

Supplemental Figure 4. Study Flow Diagram.

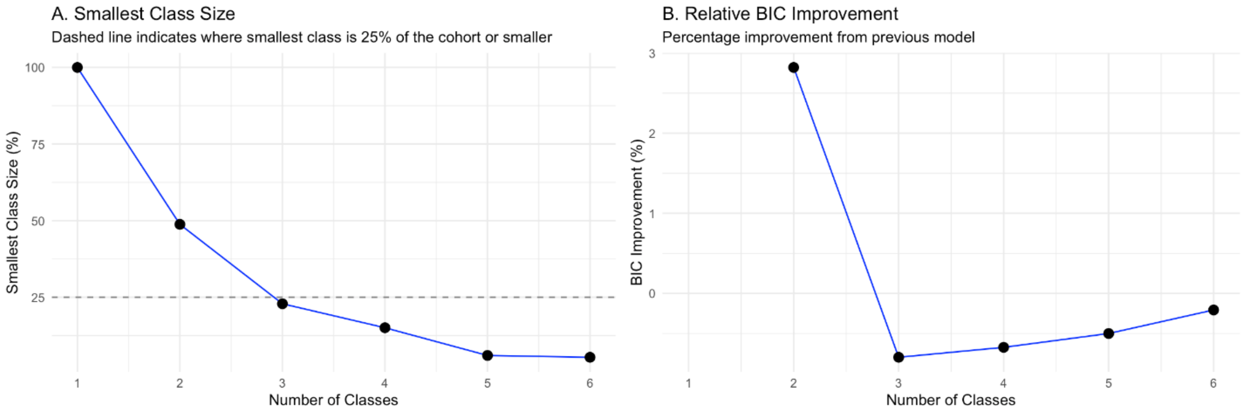

Supplemental Figure 5. Model Selection Metrics for Latent Class Analysis. (A) Size of smallest class as a percentage of total cohort. The dashed line at 25% represents a threshold for potentially unstable small class sizes. (B) Relative BIC improvement shown as percentage change from previous model, demonstrating largest improvement with the 2-class solution.

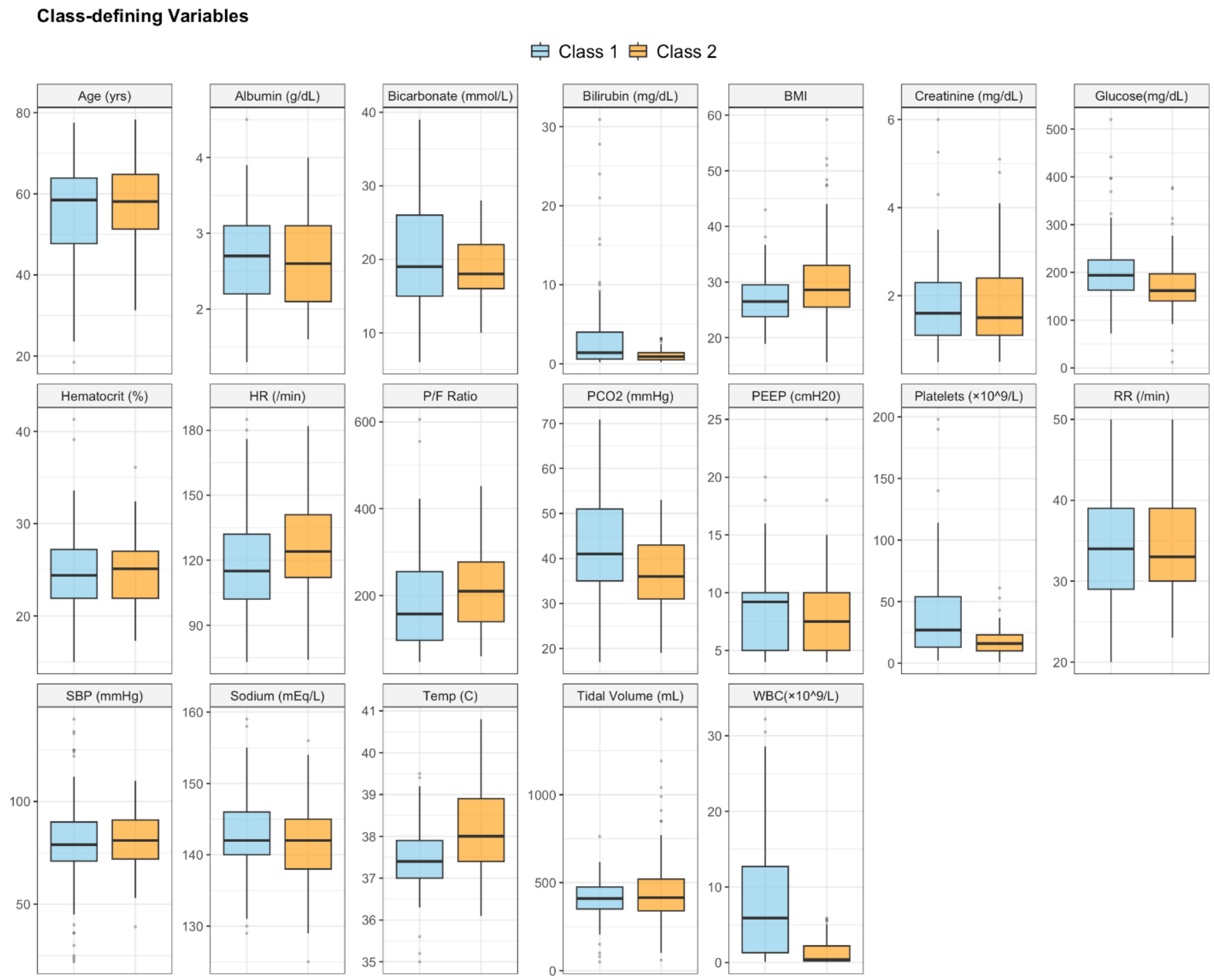

Supplemental Figure 6. Class-Defining Variables. Box plots comparing the distribution of clinical variables at time of ARDS diagnosis between Class 1 (blue) and Class 2 (orange). Variables include demographics (age, BMI), vital signs (temperature, heart rate, respiratory rate, systolic blood pressure), laboratory values (albumin, bicarbonate, bilirubin, creatinine, glucose, hematocrit, platelets, sodium, white blood cell count), and respiratory parameters (P/F ratio, PCO2, PEEP, tidal volume). The boxes show interquartile range with median line, whiskers extend to 1.5×IQR, and points represent outliers. Notable differences include lower P/F ratios, higher PCO2, and higher WBC counts in Class 1 compared to Class 2.

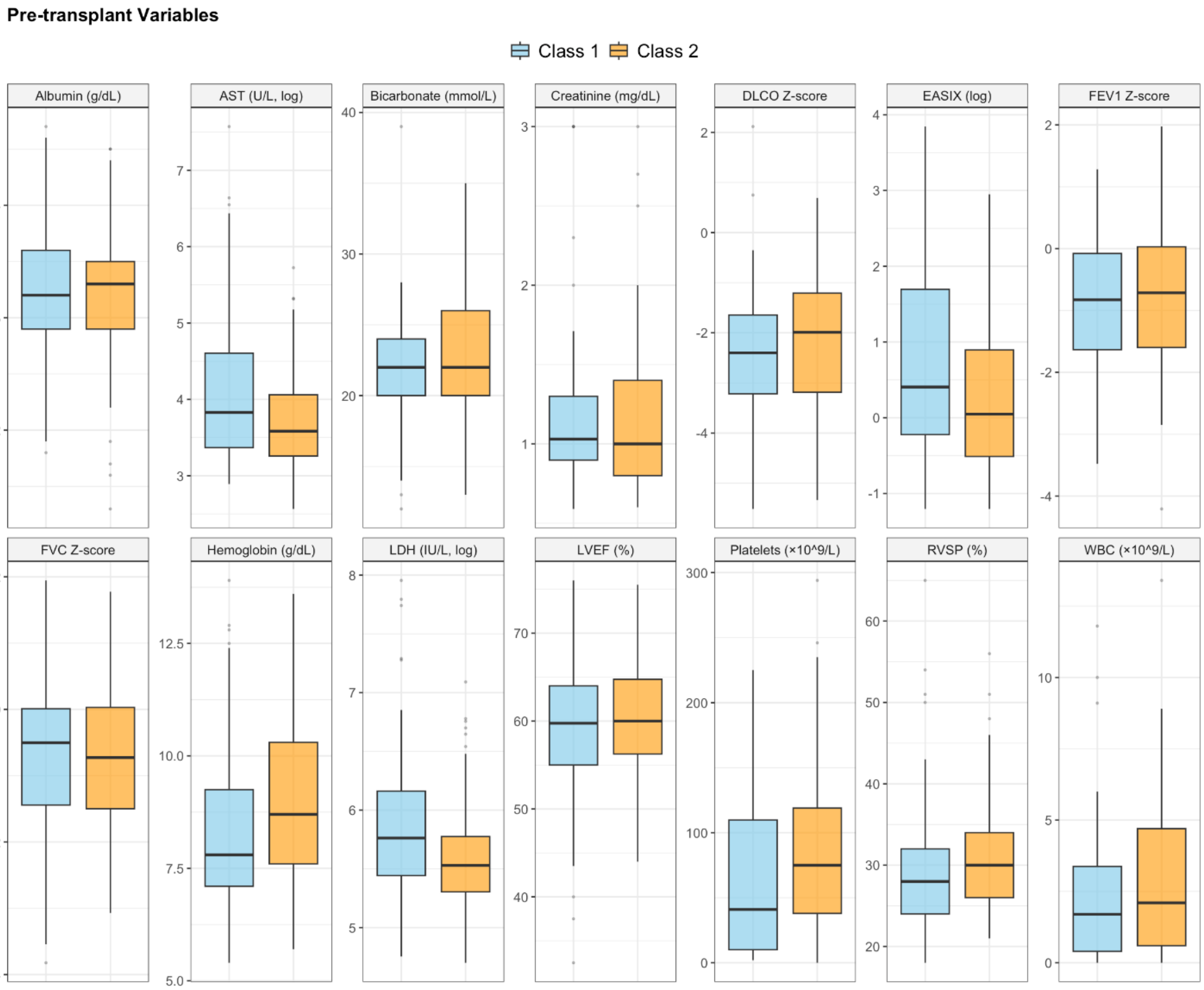

Supplemental Figure 7. Pre-Transplant Variables by Class. Box plots comparing the distribution of pre-transplant clinical variables between Class 1 (blue) and Class 2 (orange). Variables include laboratory values (albumin, AST, bicarbonate, creatinine, hemoglobin, LDH, platelets, WBC), cardiac function (LVEF), pulmonary function tests (DLCO Z-score, FEV1 Z-score, FVC Z-score, RVSP), and risk scores (EASIX). Notable differences include lower DLCO Z-scores and higher EASIX scores in Class 1, suggesting greater pre-transplant organ dysfunction in this group.

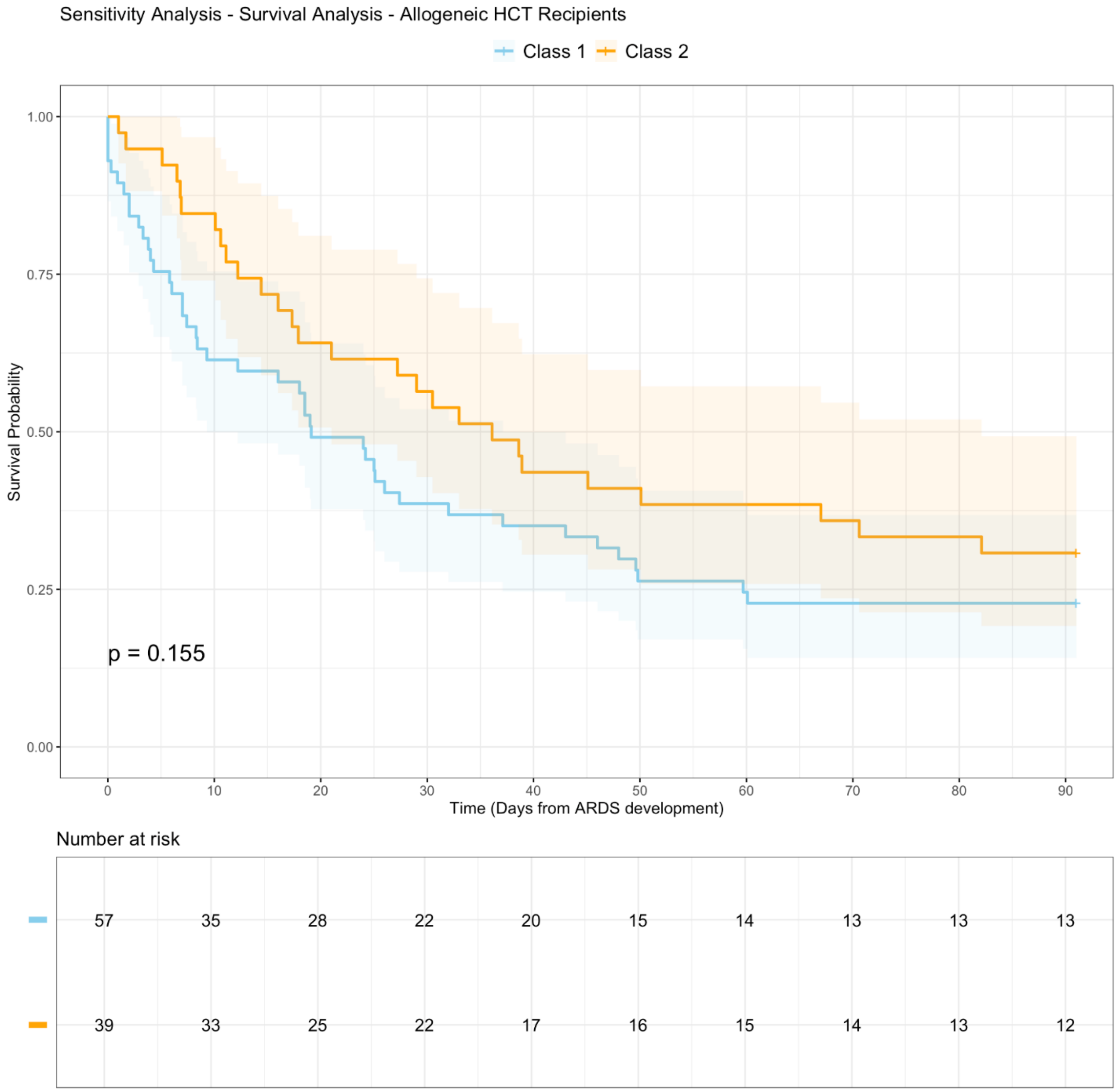

Supplemental Figure 8. Sensitivity Analysis: Survival in Allogeneic Transplant Recipients. Kaplan-Meier survival curves comparing Class 1 (blue) and Class 2 (orange) among allogeneic HCT recipients who developed ARDS. Shaded areas represent 95% confidence intervals. The number of patients at risk is shown below the graph at 10-day intervals. While Class 1 showed numerically worse survival, the difference did not reach statistical significance in the allogeneic subgroup ($p = 0.155$).

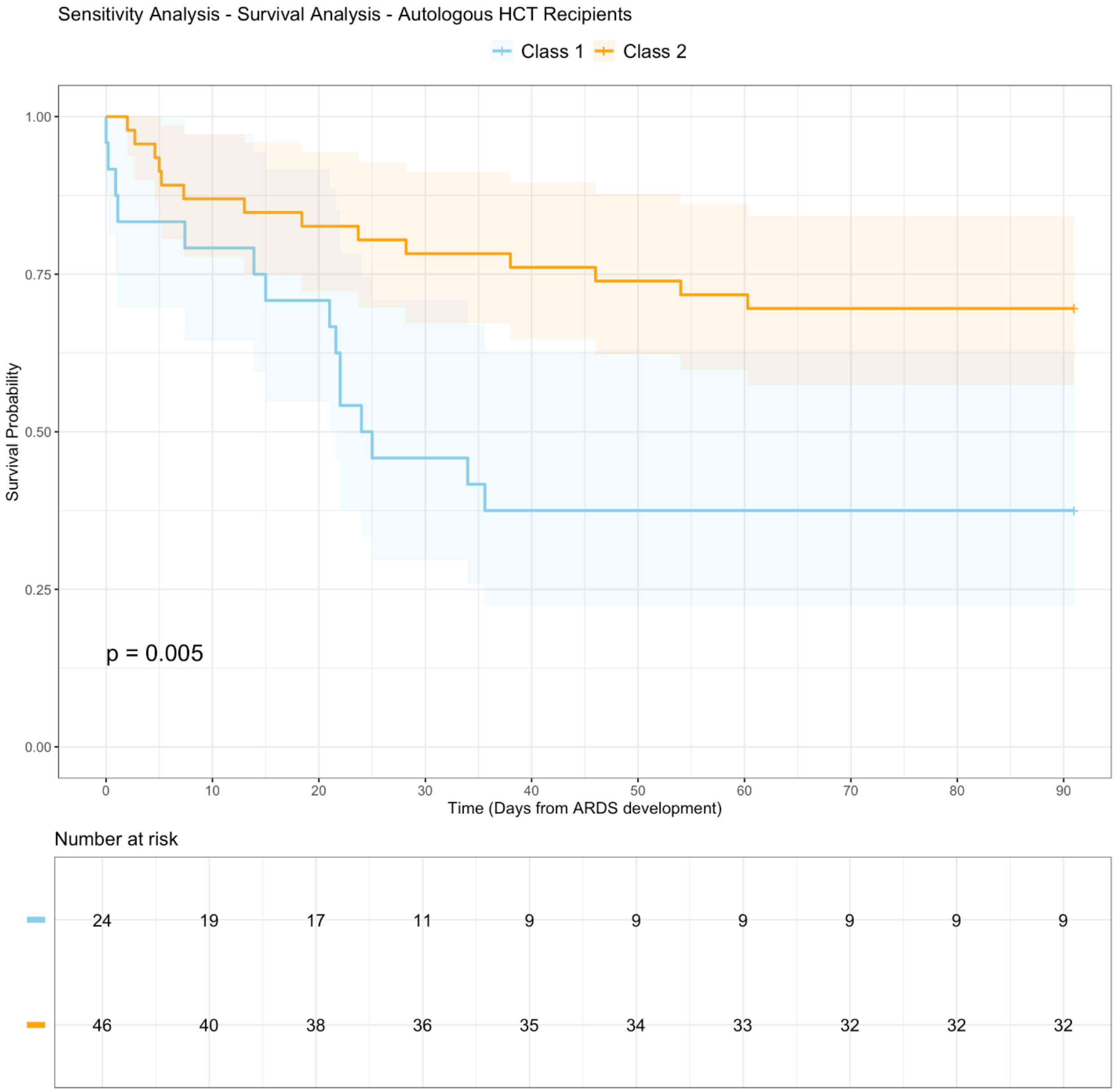

Supplemental Figure 9. Sensitivity Analysis: Survival in Autologous Transplant Recipients. Kaplan-Meier survival curves comparing Class 1 (blue) and Class 2 (orange) among autologous HCT recipients who developed ARDS. Shaded areas represent 95% confidence intervals. The number of patients at risk is shown below the graph at 10-day intervals. Class 1 demonstrated significantly worse survival compared to Class 2 in the autologous subgroup ($p = 0.005$).

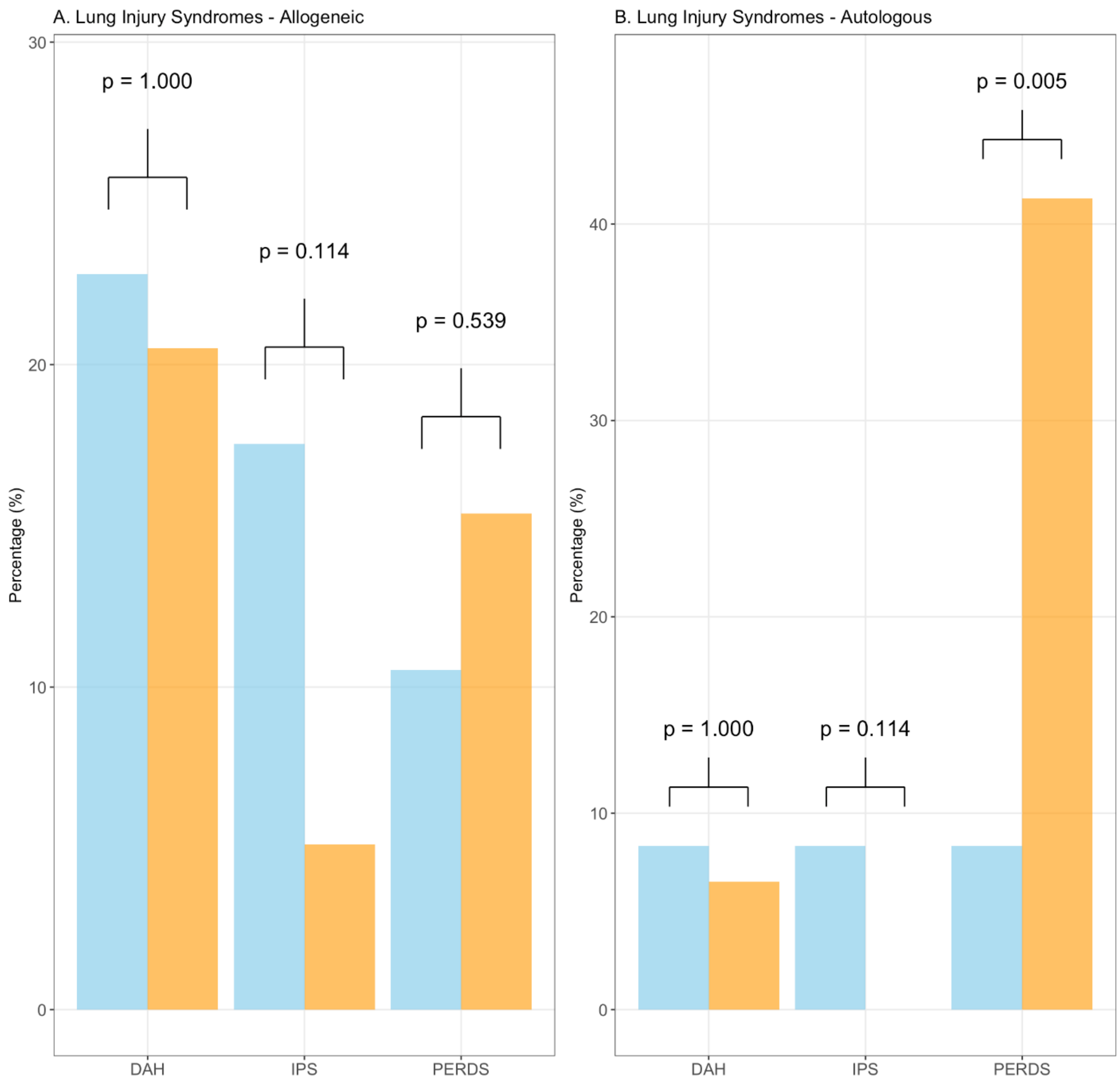

Supplemental Figure 10. Distribution of lung injury syndromes by transplant type and ARDS phenotype. Bar plots showing the frequency of specific lung injury syndromes (DAH: diffuse alveolar hemorrhage, IPS: idiopathic pneumonia syndrome, PERDS: peri-engraftment respiratory distress syndrome) in Class 1 (blue) and Class 2 (orange), stratified by transplant type. Panel A shows allogeneic HCT recipients, while Panel B shows autologous HCT recipients. P-values for between-class comparisons are shown above each pair of bars. PERDS was significantly more common in Class 2 among autologous recipients ($p = 0.005$).
